# Editorial: Mechanisms of early intracellular signaling in T lymphocytes

**DOI:** 10.3389/fimmu.2025.1638330

**Published:** 2025-06-17

**Authors:** Enrique Aguado, Mikel M. Arbulo-Echevarria, Ewoud B. Compeer

**Affiliations:** ^1^ Institute of Biomedical Research Cadiz (INIBICA), Cádiz, Spain; ^2^ Department of Biomedicine, Biotechnology and Public Health (Immunology), University of Cádiz, Cádiz, Spain; ^3^ Biogipuzkoa Health Research Institute, Molecular Oncology Group, San Sebastián, Spain; ^4^ Nuffield Department of Orthopaedics, Rheumatology and Musculoskeletal Sciences, Kennedy Institute of Rheumatology, University of Oxford, Oxford, United Kingdom

**Keywords:** TCR, T lymphocytes, thymic development, adaptor proteins, T cell activation, CAR-T signaling

T cells are central orchestrators of adaptive immunity, initiating antigen-specific responses that protect against infection and maintain immune surveillance. They recognize antigens via precise interactions between their T cell receptors (TCRs) and peptide-major histocompatibility complexes (pMHCs) on antigen-presenting cells (APCs). These interactions trigger intracellular signaling cascades that regulate T cell development, activation, and effector function.

The tight regulation of early TCR signaling is essential for mounting effective immune responses while preventing excessive or chronic activation. Upon antigen recognition, the Src-family kinase Lck is recruited to the TCR complex—particularly the CD3ε subunit—and phosphorylates ITAM motifs in the CD3 subunits. This creates docking sites for ZAP70, which activates itself and promotes downstream signaling, including LAT signalosome assembly (1). Disruption of LAT signalosome kinetics can impair T cell activation and development (2, 3). Along the same line, endocytic trafficking further shapes TCR signaling outcomes. Flotillin-mediated recycling affects nanoclustering of surface TCRs, promoting sustained signaling (4). In contrast, ESCRT proteins HRS and STAM2 mediate TCR release in extracellular vesicles via ectocytosis, contributing to signal termination (5). Thus, early TCR signaling involves key kinases (Lck, Fyn, ZAP70), phosphatases (CD45), adaptor proteins (LAT), and trafficking regulators (Flotillin, HRS, STAM), which together modulate signal strength and duration to fine-tune T cell responses (6). This Research Topic includes two review articles and two original research papers, exploring recent advances in early intracellular TCR signaling with emphasis on activation mechanisms and negative regulation.

Qin and Xu review how TCR signaling strength, duration, and integration with co-stimulation, cytokines, and metabolic cues influence T cell lineage decisions. For instance, they discuss recent work that found strong TCR signals to suppress the transcription factor KLF2, essential for maintaining effector lineage fidelity. Genetic deletion of KLF2 skews cells toward an exhausted-like phenotype. Their review also highlights TCR-driven control of Treg versus Th17 fate: robust signaling promotes Th17 differentiation via Lck/Fyn-STAT3 and MALT1-mediated cleavage of Roquin and Regnase-1—post-transcriptional repressors of Th17 genes. In contrast, engineered TCRs with non-signaling ITAMs reduce signaling strength and favor Treg differentiation, emphasizing how TCR signal tuning influences immune balance.

Along these lines, Love et al. present a comprehensive review of ITAM function, highlighting three primary models: signal discrimination, signal amplification, and signal duality. Recent studies, including their own, show that certain ITAMs can deliver both activating and inhibitory signals depending on pMHC affinity and subunit context. This duality contributes to ligand discrimination and immune modulation, offering a mechanistic explanation for ligand-mediated antagonism—a long-standing mystery in TCR biology. These insights support a more refined view of TCR regulation and may inform strategies for enhancing or suppressing T cell responses.

Calcium (Ca²^+^) fluxes are critical regulators of T cell activation, differentiation, and fate decisions. In this context, Schreiber et al. explore the role of anoctamin 9 (ANO9), a Ca²^+^-activated chloride channel, in regulating TCR-induced Ca²^+^ signaling. Ca²^+^ entry is mainly mediated by store-operated ORAI1 channels, coordinated by STIM1 and PIP^2^. ANO9 helps recruit Ca²^+^-ATPases to the plasma membrane, maintaining optimal Ca²^+^ levels near ORAI1 channels and preventing inhibition of store-operated Ca²^+^ entry (SOCE). This positions ANO9 as a critical modulator of early TCR signaling. Functional studies in Jurkat T cells and primary lymphocytes confirm its role in Ca²^+^ signal initiation, suggesting ANO9 as a potential target for tuning T cell activation in immune disorders.

Kim et al. present original research on ARAP, a novel adaptor protein implicated in TCR signaling and integrin-mediated adhesion. ARAP-deficient T cells exhibit impaired phosphorylation of key signaling molecules, including PLC-γ1, SLP-76, Akt, and ERK, resulting in reduced activation, proliferation, and cytokine production. In a mouse model of experimental autoimmune encephalomyelitis (EAE), ARAP-deficient mice show lower IFN-γ levels and milder disease, underscoring ARAP’s essential role in T cell-mediated immune responses. This protein emerges as a promising candidate for therapeutic modulation in autoimmunity.

While TCR signaling has been studied for decades, it continues to yield new insights that reshape our understanding of T cell regulation. The studies featured in this Research Topic illuminate critical components of early TCR signaling: the dual roles of ITAMs in fine-tuning signal output, ANO9’s control of Ca²^+^ dynamics, and ARAP’s integration of TCR and adhesion signals. Each discovery offers new therapeutic angles for immune modulation.

These findings collectively underscore the complexity and plasticity of TCR-proximal signaling. Feedback mechanisms, signal modulators, and membrane trafficking elements all converge to calibrate TCR responses. Such precision is key not only for immune homeostasis but also for effective interventions in cancer, infection, and autoimmunity.

Moving forward, unraveling how these signaling pathways are integrated will be critical for understanding T cell fate decisions and for developing precision immunotherapies. Mechanistic insights into early TCR events may enable the rational design of enhanced CAR-T cells, novel checkpoint inhibitors, and modulators that preserve immune balance while boosting specific responses—for example, against tumors.
